# The Association of Sarcopenic Obesity With Respiratory Function Outcomes in Adults: A Systematic Review and Meta‐Analysis

**DOI:** 10.1111/obr.70157

**Published:** 2026-05-08

**Authors:** Tasneem Baabbad, Magaly Aceves‐Martins, Graham Horgan, Phyo Kyaw Myint, Baukje de Roos

**Affiliations:** ^1^ The Rowett Institute, School of Medicine, Medical Sciences and Nutrition University of Aberdeen Aberdeen UK; ^2^ Biomathematics and Statistics Scotland University of Aberdeen Aberdeen UK; ^3^ Institute of Applied Health Sciences, School of Medicine, Medical Sciences and Nutrition University of Aberdeen Aberdeen UK

**Keywords:** obesity, older population, respiratory functions, sarcopenia

## Abstract

Sarcopenic obesity has been associated with various health conditions and mortality. Our objective was to systematically review and estimate the association between sarcopenic obesity and respiratory function outcomes. We searched EMBASE, MEDLINE, and Google Scholar for observational studies involving adults (≥ 18 years), defined sarcopenic obesity, and assessed respiratory outcomes in those with and without sarcopenic obesity. A random‐effects meta‐analysis was conducted for the outcomes of forced vital capacity (FVC), forced expiratory volume in 1 s (FEV1), and the ratio of forced expiratory volume in 1 s to forced vital capacity (FEV_1_/FVC). Seven papers representing 173,677 participants were included. The risk of bias assessment using the JBI Critical Appraisal Checklist revealed that all studies had a low risk of bias. The meta‐analysis included data from five studies, revealing a significant association between sarcopenic obesity and lower FVC (in L) (−0.77; 95% CI, −1.27, −0.26) and FEV1 (in L) (−0.84; 95% CI, −1.26, −0.42), both with high heterogeneity (*I* ^2^ = 94%). Sarcopenic obesity was significantly associated with respiratory function outcome (FEV1) when obesity was assessed using fat mass % (−1.14; 95% CI, −1.21, −1.06), but not when using the body mass index (BMI). In conclusion, sarcopenic obesity was significantly associated with poorer respiratory function outcomes. Assessment of respiratory function may be desirable in those with sarcopenic obesity, especially in high‐risk populations such as older adults.

**Abbreviations**
6MWT: six‐minute walk test
ASM: appendicular skeletal muscle mass
BMI: body mass index
CAT: the COPD Assessment Test
COPD: chronic obstructive pulmonary disease
DLCO: maximum diffusing capacity of the lung
FEV1: forced expiratory volume in the first second
FEV1/FVC: the ratio of forced expiratory volume in the first second to forced vital capacity
FFM: fat‐free mass
FVC: forced vital capacity
GOLD: The Global Initiative for Obstructive Lung Disease
HRQoL: health related quality of life
LMM: low muscle mass
MEF: maximal mid‐expiratory flow rate
mMRC: modified Medical Research Council scale
PEF: peak expiratory flow
SGRQ: St. George's Respiratory Questionnaire
SMMI & SMI: skeletal muscle mass index
TNFa: tumor necrosis factor alpha
UK: United Kingdom
WC: waist circumference

## 1 Introduction

Sarcopenia, characterized by the loss of muscle mass as well as strength, is an age‐related syndrome that still has no broadly accepted clinical definition [1]. Previous studies have used various variables to assess sarcopenia, including the measurement of muscle mass, muscle strength, and physical performance [1]. It can be a risk factor for other health conditions, including disability, metabolic diseases, as well as other comorbidities and mortality [2, 3]. For example, sarcopenia and skeletal muscle dysfunction are prevalent in patients with respiratory disease, including those with chronic obstructive pulmonary disease (COPD), affecting their functional capacity and mortality [4]. The prevalence of sarcopenia in patients with COPD is high, ranging from 8% in population‐based studies to 21% in clinic‐based studies and to 63% in patients residing in nursing homes [5]. Notably, low muscle mass in COPD patients has been associated with reduced respiratory function, exercise capacity, and muscle strength, especially in patients who also had overweight and obesity [6, 7]. The association between low muscle mass and strength, and reduced respiratory function, is clinically relevant, as reduced forced expiratory volume in the first second (FEV1) is a marker of cardiovascular mortality [8], and has been linked to higher mortality rates in the general population [8, 9].

Concurrently, older populations also have a higher risk of developing obesity and an increase in fat mass [10]. An increase in adipose tissue is believed to enhance levels of pro‐inflammatory cytokines, such as tumor necrosis factor‐alpha (TNFα), which could subsequently reduce muscle strength [11]. Age‐related changes in body composition often include both increased fat mass and decreased muscle mass. This combination is referred to as sarcopenic obesity, which frequently occurs in older adults [12]. According to data from the United Kingdom (UK) Biobank, the prevalence of sarcopenic obesity is estimated to be 4% in older adults, 2.8% in women, and 1.2% in men [13]. Sarcopenic obesity can lead to an increased risk of other health conditions including cardiovascular disease, diabetes, and physical disability, compared to those who have obesity or sarcopenia alone [13, 14, 15]. Sarcopenic obesity was also reported to be a risk factor for respiratory disease outcomes and worsening lung function [16]. Changes in body composition have been linked to respiratory impairment observed in patients with COPD [17]. Interestingly, the coexistence of sarcopenia and obesity made individuals significantly more likely to have severe COVID‐19, potentially as a result of reduced respiratory functioning, a compromised immune response, and a decreased ability to respond to metabolic stress [18]. The underlying mechanisms for muscle dysfunction in COPD may involve systemic inflammation, oxidative stress, and anabolic/catabolic hormone imbalance [4]. Sarcopenic obesity has been linked to increased levels of inflammatory biomarkers, including C‐reactive protein (CRP), interleukin‐6 (IL‐6), and its soluble receptor (sIL‐6R) [11]. A concurrent decrease in anti‐inflammatory cytokines may additionally compromise respiratory and musculoskeletal health [19].

Although there is increasing evidence of the association between sarcopenic obesity and impaired respiratory outcomes, the characteristics and intensity of this relationship remain unclear. This systematic review and meta‐analysis aims to investigate the association between the prevalence of sarcopenic obesity and respiratory function outcomes.

## 2 Methods

This systematic review followed the Preferred Reporting Items for Systematic Reviews and Meta‐Analyses (PRISMA) guidelines [20]. A proposal for this study was prospectively registered with the International Prospective Register of Systematic Reviews (PROSPERO) [21], registration number CRD42024518254. The systematic review strategy was based on the Population, Exposure, Comparator, Outcomes, and Study (PECOS) design framework (Table 1).

**TABLE 1:** PECOS framework.

Population	Adults aged 18 years and over, all genders, all ethnicities
Exposure	Sarcopenic obesity, body composition, muscle mass decrease and fat mass increase, or other related terms.
Comparator	Studies comparing participants with sarcopenic obesity versus those without sarcopenic obesity
Outcomes	Prevalence of respiratory outcomes such as forced vital capacity (FVC), forced expiratory volume in the first second (FEV1), and the ratio of forced expiratory volume in the first second to forced vital capacity (FEV_1_/FVC).
Study design	Observational studies

### 2.1 Search Strategy and Selection Criteria

Searches were conducted in MEDLINE (from 1946 to January 2024) and EMBASE (from 1974 to January 2024), and an updated search was conducted in July 2024. The search strategy included a combination of keywords and MeSH terms, such as “sarcopenia” OR “loss of muscle mass”, “obesity” OR “fat mass”, OR “sarcopenic obesity”, AND “respiratory disease” OR “impaired lung function”. We also searched using subject terms and truncation symbols. References of included studies were manually screened to identify additional studies. Additionally, we used the search engine Google Scholar to identify relevant gray literature or potentially missed studies. The full search strategy is shown in Table S1.

### 2.2 Inclusion and Exclusion Criteria

Studies were included if (1) participants were aged ≥ 18 years old (no sex, race, or geographical restrictions), (2) the study defined sarcopenic obesity (either using the term specifically or by measuring loss in muscle mass and increase in fat mass, based on cut‐off values), (3) the study measured the prevalence of both sarcopenic obesity and absence of sarcopenic obesity, (4) the study measured respiratory function in both participants with sarcopenic obesity and in participants without sarcopenic obesity, and (5) studies were published in the English language.

We excluded studies that (1) included children and young people under 18 years old, (2) measured sarcopenic obesity as an outcome and respiratory function as an exposure, (3) did not define sarcopenic obesity, and/or only used the term “change in body composition” without including cut‐off points to define sarcopenic obesity, (4) were not primary research, or were systematic reviews, case reports, protocols, animal experiments, and conference summaries, or (5) were not written in the English language.

### 2.3 Study Selection

One researcher (TB) reviewed the studies by screening each title and abstract. A second reviewer (MA‐M) independently screened 10% to check for consistency. Two researchers (TB and MA‐M) screened the full text to confirm inclusion or exclusion criteria if the title and abstract were unclear. A third investigator (BdR) would check against inclusion and/or exclusion criteria in case of disagreement between the first two reviewers.

### 2.4 Data Extraction

Two researchers (TB and MA‐M) independently extracted data from the included studies and entered them into a spreadsheet. All the relevant data were extracted using a data extraction form developed explicitly for this review, following the PECOS framework (see Table 1). The form included information on the author, year, country of study, and study design including sample size, age, and sex of participants, type of population, sarcopenic obesity definition and cut‐off points, sarcopenic obesity prevalence, respiratory function measurements, and measurements used to assess the association between exposure and outcome.

### 2.5 Data Synthesis and Analysis

A narrative synthesis was conducted across all included studies, and the main characteristics of the studies were tabulated. Studies that reported the prevalence of sarcopenic obesity and the mean and standard deviations (SD) of respiratory function outcomes in both groups were included, and data were extracted for the meta‐analysis. For studies that used standard errors (SE), data were transformed to SD by multiplying the value by the square root of the sample size. Heterogeneity was assessed using I^2^ statistics, and a random‐effects model was employed for this analysis. To examine the robustness of the results across various definitions of obesity and to evaluate potential differences in respiratory outcomes, we performed a sensitivity analysis using the different methods applied across studies to diagnose obesity. For this, we specifically conducted a meta‐analysis stratified by a definition for obesity (i.e., studies that defined obesity using BMI (kg/m^2^) vs. those using body fat percentage). Additionally, we compared FEV1 (in L) between participants with and without sarcopenic obesity to assess whether the observed associations remained consistent across these subgroups. The analysis was conducted using R, with the “meta” library [22].

### 2.6 Assessment of Risk Bias

Two researchers (TB and MA‐M) independently evaluated the methodological quality of the cross‐sectional studies using the JBI Critical Appraisal Checklist for Analytical Cross‐Sectional Studies [23].

## 3 Results

From the search, 768 references were identified, from which 104 duplicate records were removed, and 664 titles and abstracts were screened. As a result, 605 records were excluded, and 23 records were not retrieved. Thirty‐six studies were assessed for eligibility, and 29 studies were excluded. Seven cross‐sectional studies [24, 25, 26, 27, 28, 29, 30] were included for a narrative synthesis, and five of these were used for a meta‐analysis (Figure 1) [24, 25, 26, 27, 29]. The seven studies included 173,677 participants aged 18 years and older, recruited from five countries: China, Korea, Mexico, the United Kingdom, and the Netherlands. Most studies used data from different databases (*n* = 6), while one study was conducted in a clinical setting (Table 2). Participants were both men and women, except for one study, which was conducted only in men [25]. Studies employed various methodologies to assess outcomes related to sarcopenia, obesity, muscle mass, and respiratory function. Some studies defined sarcopenia using multiple methods, while others used only one measurement (Table 2). Six studies measured muscle mass, calculated by dividing appendicular skeletal muscle mass (ASM) in kg, by either height squared in meters (m^2^) [24, 26, 27, 29] or weight in kg × 100 [25, 28]. One study assessed muscle mass from a deviation of 24‐h urinary excretion of creatinine of ≤ 1.0 SD from the sex‐specific mean [30]. Three studies also used handgrip strength [24, 26, 27], and one used poor physical performance to define sarcopenia [24]. Obesity was defined by three measurements: fat mass [26, 27, 29], body mass index (BMI) [24, 25, 27, 28, 30], and waist circumference [27]. Six studies measured respiratory function by assessing FVC (L), FVC (%) [25, 26, 28, 29], FEV1 (L), FEV1 (%) [24, 25, 26, 27, 28, 29], and/or the ratio of FEV_1_/FVC (%) [24, 25, 28, 29]. Studies also applied other tests to measure respiratory outcomes, including the modified Medical Research Council scale (mMRC) to measure symptoms of dyspnea [24], four types of St. George's Respiratory Questionnaire (SGRQ) to assess Health‐Related Quality of Life (HRQoL) [24], the COPD Assessment Test (CAT) to measure the quality of life [24], the Global Initiative for Obstructive Lung Disease (GOLD 1 to 4) [25], Maximal Mid‐Expiratory Flow rate (MEF) [26], Peak Expiratory Flow (PEF) [26], Maximum Diffusing Capacity of the Lung (DLCO) [26], the Six‐Minute Walk Test (6MWT) [26], respiratory incidence and mortality [27], and COVID‐19 hospitalization [30]. The adjustment strategies differed between studies, as each included different covariates in their models (Table 2). All studies controlled for age and sex [25, 26, 27, 28, 29, 30], while some also adjusted for smoking status [25, 27, 28, 29], comorbidities [26, 27, 29] and morbidities [30], BMI [25, 28], alcohol consumption [27, 28, 29], physical activity [27, 28], or socioeconomic factors [27, 30]. One study did not adjust for any covariates when examining the association between sarcopenic obesity and lung function outcome [24], increasing the likelihood of residual confounding, and this must be taken into account when interpreting the aggregated results.

**FIGURE 1:**
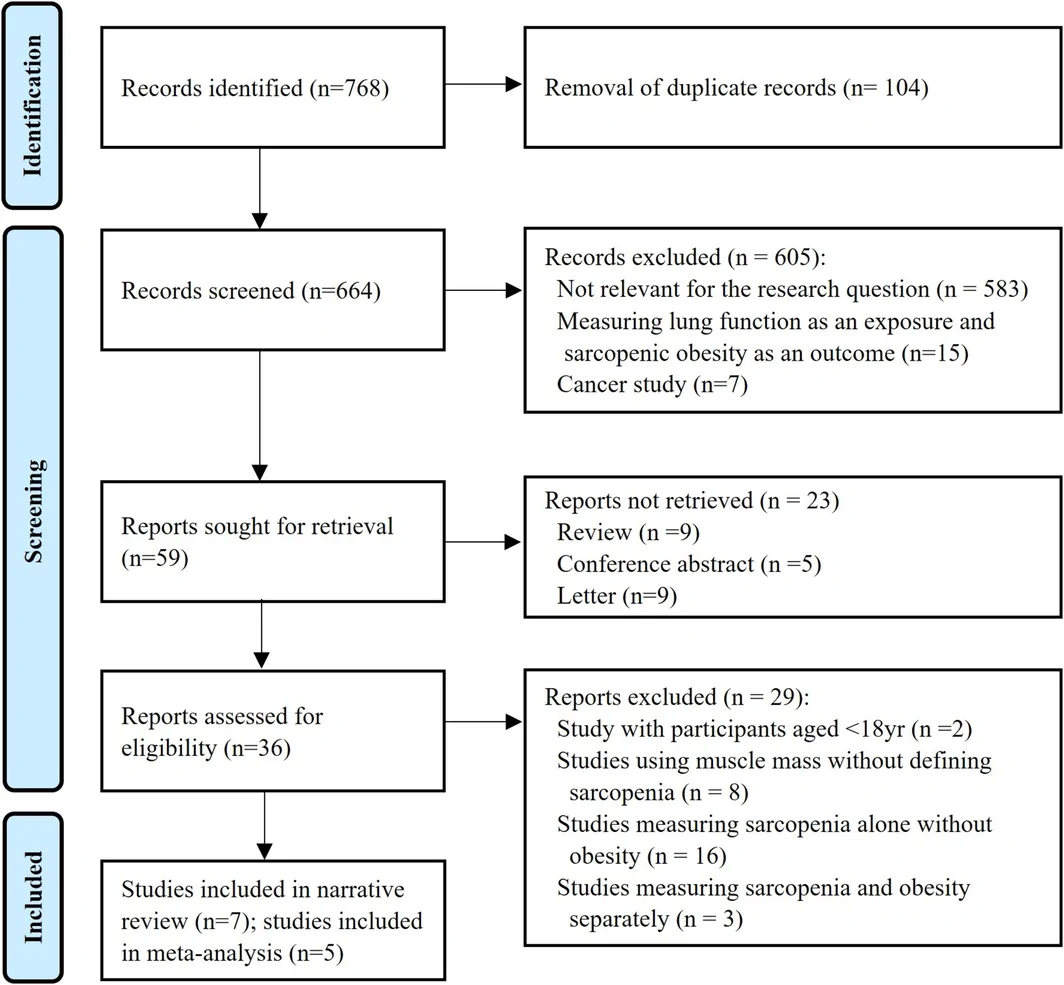
PRISMA flowchart.

**TABLE 2:** Characteristics of selected studies in the systematic review.

References	Country, sample size and setting	Age	Sex *n* (%)	Tools to measure sarcopenic obesity muscle mass	Sarcopenic obesity cut‐off points	Sarcopenic obesity prevalence *n* (%)	Respiratory disease measurements	Covariates
Wang et al. [24]	China, 198, Clinical setting	> 18 years	Male 140 (70.7%) Female 58 (29.3%)	**Sarcopenia** SMMI ^a^ Handgrip strength Poor physical performance **Obesity** BMI **Muscle mass** BIA	**Sarcopenia** Males SMMI < 7.0 kg/m^2^ Handgrip strength < 28 kg Poor physical performance ≥ 12 s Females SMMI < 5.7 kg/m^2^ Handgrip strength < 18 kg Poor physical performance ≥ 12 s **Obesity** BMI ≥ 25.0 kg/m^2^	19 (9.6%)	FEV1 (%) FEV_1_/FVC (%) mMRC to measure symptoms of dyspnoea CAT (quality of life) SGRQ (HRQoL, activity score) SGRQ (HRQoL, impact score) SGRQ (HRQoL symptom score) SGRQ (HRQoL total score)	None
Koo et al. [25]	Korea, 574, Population‐based	≥ 40 years	All male 574	**Sarcopenia** SMI (%)^b^, ^c^ **Obesity** BMI **Muscle mass** DEXA	**Sarcopenia** SMI ≤ 29.8% **Obesity** BMI ≥ 25.0 kg/m^2^	73 (14.2%)	Stages of COPD GOLD 1–4 FVC (L & %) FEV1 (L & %) FEV_1_/FVC (%)	Age, sex, BMI, smoking
Orea‐Tejeda et al. [26]	Mexico, 711, Population‐based	54 ± 14 years^e^	Male 455 (64%) Female 256 (36%)	**Sarcopenia** ASM^a^ Handgrip strength **Obesity** Fat mass **Muscle mass** BIA	**Sarcopenia** Males ASM < 7.0 kg/m^2^ Handgrip strength < 27 kg Females ASM < 6.0 kg/m^2^ Handgrip strength < 16 kg **Obesity** Fat mass < 25%	27 (3.8%)	FEV1 (L & %) FVC (L & %) MEF 25%–75% PEF DLCO (%) 6MWT (mts)	Age, sex, comorbidities
Petermann‐Rocha et al. [27]	UK, 170,083 Population‐based	≥ 65 years	Male 57,469 (41.4%) Female 81,085 (58.6%)	**Sarcopenia** Grip strength Muscle mass index^a^ **Obesity** BMI Fat mass WC **Muscle mass** BIA	**Sarcopenia** Males Grip strength < 30 kg Muscle mass index < 8.87 kg/m^2^ Females Grip strength < 20 kg Muscle mass index < 6.42 kg/m^2^ **Obesity** Males Fat mass ≥ 26.9% WC ≥ 102 cm BMI ≥ 30 kg/m^2^ Females Fat mass ≥ 38.6% WC ≥ 88 cm BMI ≥ 30 kg/m^2^	3509 (18.2%)	FEV1 (L) Respiratory incidence Respiratory mortality	Age, sex, gross income, education attainment, comorbidities, smoking, sleep duration, physical activity, total discretionary sedentary time, dietary intake, and alcohol consumption
Moon et al. [28]	Korea, 1583, Population‐based	≥ 65 years	Male 635 (40%) Female 948 (60%)	**Sarcopenia** LMM^b^, ^d^ **Obesity** BMI **Muscle mass** DEXA	**Sarcopenia** Males LMM ≤ 27.85% Females LMM ≤ 21.35% **Obesity** BMI > 25 kg/m^2^	Males 50 (7.8%) Females 91 (9.6%)	FVC (L & %) FEV1 (L & %) FEV_1_/FVC (%)	Age, sex, BMI, smoking, physical activity, alcohol consumption
Lim et al. [29]	Korea, 26,343, Population‐based	≥ 50 years	Male 8640 (32.8%) Female 17,703 (67.2%)	**Sarcopenia** SMI^a^ **Obesity** Fat mass **Muscle mass** DEXA	**Sarcopenia** Males SMI < 7.0 kg/m^2^ Females SMI < 5.7 kg/m^2^ **Obesity** Males Body fat mass > 25% Females Body fat mass > 35%	661 (2.5%)	FVC (L & %) FEV1 (L & %) FEV_1_/FVC (%)	Age, sex, comorbidities, smoking, alcohol consumption
Zhu et al. [30]	Netherlands, 3594, Population‐based	45 ± 11 years^e^	Male 1276 (35.5%) Female 2318 (64.5%)	**Sarcopenia** muscle mass **Obesity** BMI **Muscle mass** 24‐h urinary excretion of creatinine	**Sarcopenia** A deviation of 24‐h urinary excretion of creatinine of ≤ 1.0 SD from the sex‐specific mean **Obesity** BMI > 25 kg/m^2^	140 (3.9%)	COVID‐19 hospitalization	Age, sex, being a health care worker, education level, morbidities, and lifestyle risk factors

Abbreviations: 6MWT, Six‐Minute Walk Test; ASM, Appendicular Skeletal Muscle Mass; BMI, Body Mass Index; CAT, COPD Assessment Test; DLCO, Maximum Diffusing Capacity of the Lung; FEV1 (%), Percentage of predicted value compares between the actual volume of FEV1 and an average of the normal FEV1 for a person of the same gender, height, and age; FEV1/FVC (%), The ratio of Forced Expiratory Volume in the first second to Forced Vital Capacity; FEV1 (L), Forced Expiratory Volume in the first second; FVC (%), Percentage of predicted value compares between the actual volume of FVC and an average of the normal FVC for a person of the same gender, height, and age; FVC (L), Forced Vital Capacity; GOLD, Global Initiative for Obstructive Lung Disease; HRQoL, Health‐Related Quality of Life; LMM, Low Muscle Mass; MEF, Maximal Mid‐Expiratory Flow Rate; mMRC, modified Medical Research Council scale; mts, meters; PEF, Peak Expiratory Flow; s, second; SGRQ, St. George's Respiratory Questionnaire; SMMI & SMI, Skeletal Muscle Mass Index; WC, Waist Circumference.

^a^ Calculated by dividing appendicular skeletal muscle mass (kg) by height squared in meters (m^2^).

^b^ Calculated by dividing appendicular skeletal muscle mass (kg) by weight (kg) × 100.

^c^ Less than one SD below the gender‐specific mean for a young reference group between 20 and 39 years of age based on a previous Korean cohort study.

^d^ Two SD below the sex‐specific mean of the young reference group.

^e^ Mean and SD.

All studies provided the total prevalence of sarcopenic obesity, which ranged from 2.5% to 18.2%, without distinguishing between sex or other demographic parameters [24, 25, 26, 27, 29, 30], except for one study that gave the prevalence in men and women separately [28]. A significant association between the prevalence of sarcopenic obesity and some or all of the respiratory function outcomes was reported in five studies [24, 25, 27, 28, 29], while there was no significant association observed in one study [26]. One study reported a relationship between sarcopenic obesity and respiratory function in men only [28]. A significant association was also found between sarcopenic obesity and the risk of hospitalization in those infected with COVID‐19 [30].

A meta‐analysis was conducted using cross‐sectional data from five studies to evaluate the association of sarcopenic obesity and lung function outcomes rather than causation [24, 25, 26, 27, 29]. Two studies were excluded from the meta‐analysis, as one did not provide enough information to calculate the effect size [28], and the other did not measure spirometry values as outcomes [30]. Forest plots were plotted to visualize the associations between sarcopenic obesity and five spirometry results: FVC (L), FVC (%), FEV1 (L), FEV1 (%), and FEV_1_/FVC (%) (Figures 2, 3, 4, 5, 6). Data from the same three studies were used for the meta‐analyses with FVC, FVC, and FEV1 as outcomes [25, 26, 29], including 761 participants with and 8913 participants without sarcopenic obesity (Figures 2, 3, and 5). Data from four studies were used for the meta‐analysis with FEV1 as an outcome [25, 26, 27, 29] (Figure 4), including 4270 participants with and 53,051 participants without sarcopenic obesity. Data from three studies were used for the meta‐analysis with the ratio of FEV_1_/FVC as an outcome, including 753 participants with and 8891 participants without sarcopenic obesity [24, 25, 29] (Figure 6). Sarcopenic obesity was associated with a significant reduction in FVC (in L) (−0.77; 95% CI, −1.27 to −0.26; *I* ^2^ = 94%) (Figure 2), FVC (as %) (−0.44; 95% CI, −0.60 to −0.28; *I* ^2^ = 32%) (Figure 3), FEV1 (in L) (−0.84; 95% CI, −1.26 to −0.42; *I* ^2^ = 94%) (Figure 4), and FEV1 (as %) (−0.16; 95% CI, −0.24 to −0.09; *I* ^2^ = 0%) (Figure 5). There was no significant relation between sarcopenic obesity and the ratio of FEV_1_/FVC (Figure 6).

**FIGURE 2:**
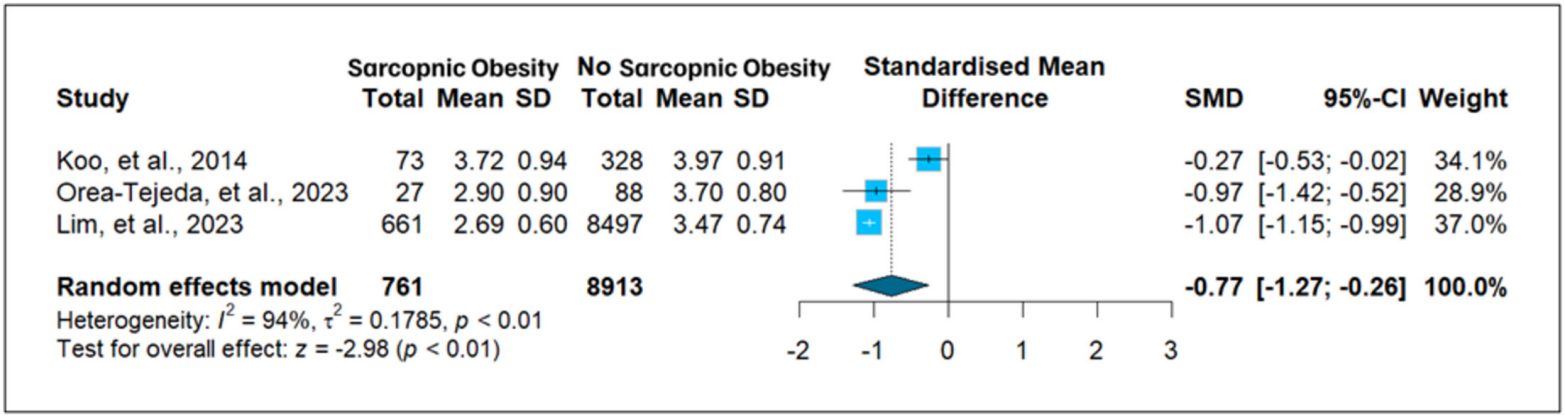
Forest plot comparing participants with and without sarcopenic obesity for Forced Vital Capacity (FVC, in L). SMD = standardized mean difference.

**FIGURE 3:**
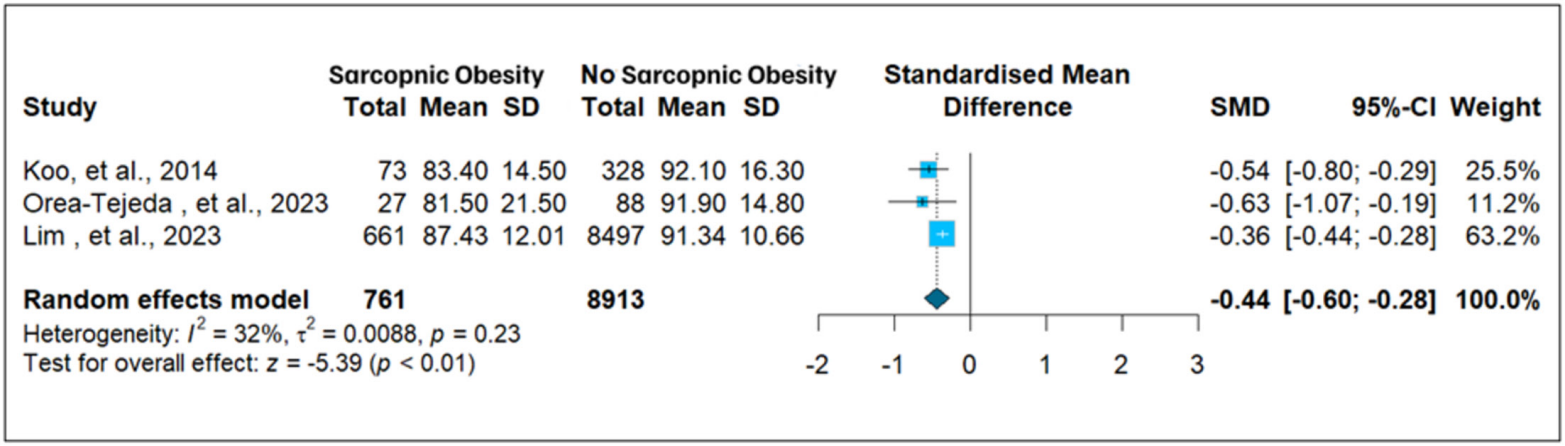
Forest plot comparing participants with and without sarcopenic obesity for Forced Vital Capacity (FVC, as %). SMD = standardized mean difference.

**FIGURE 4:**
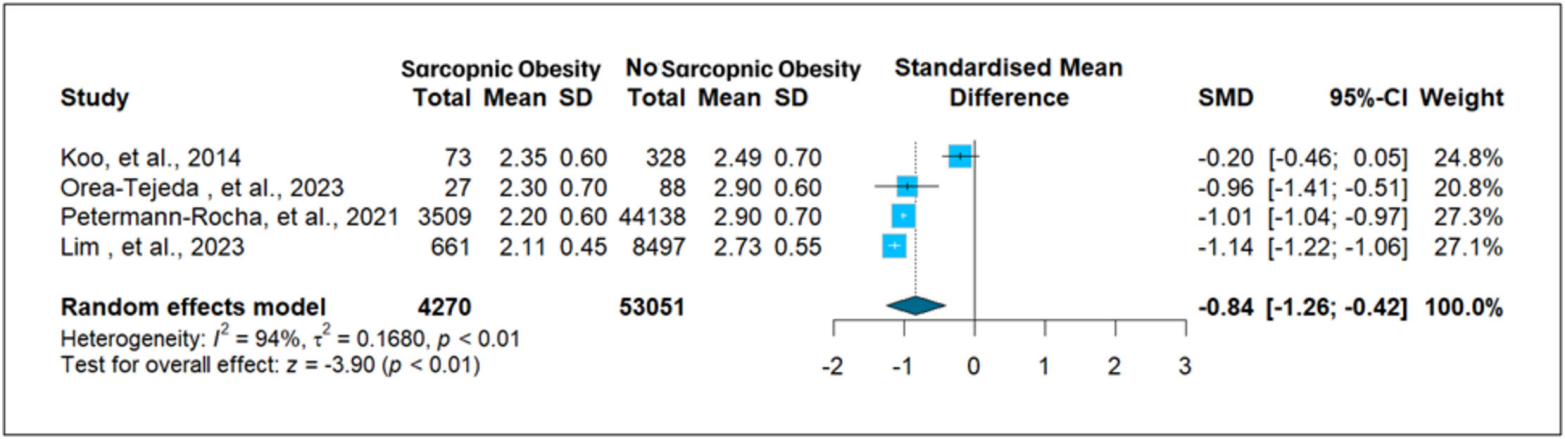
Forest plot comparing participants with and without sarcopenic obesity for Forced Expiratory Volume in the first second (FEV1, in L). SMD = standardized mean difference.

**FIGURE 5:**
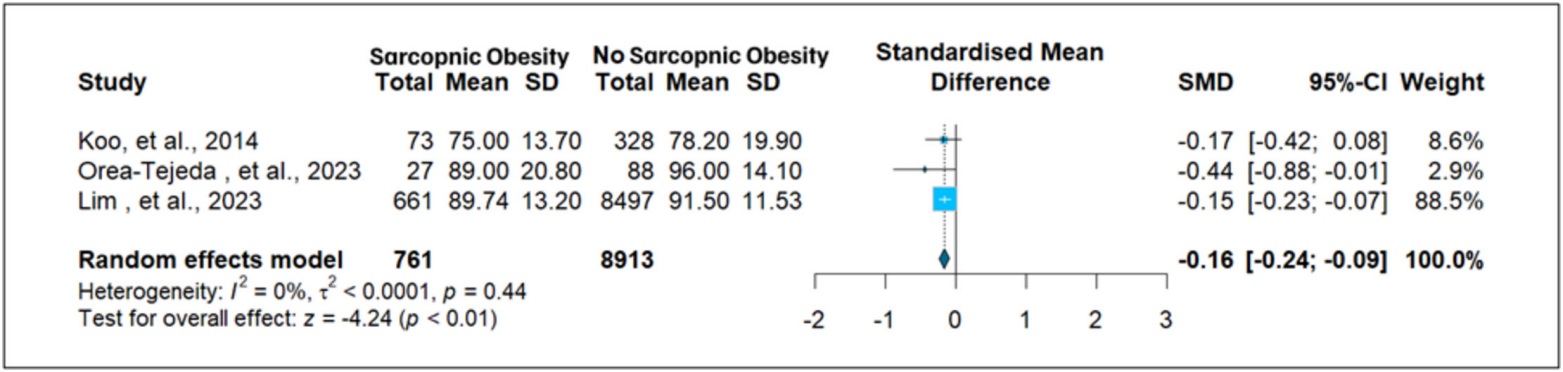
Forest plot comparing participants with and without sarcopenic obesity for Forced Expiratory Volume in the first second of the association between sarcopenic obesity and (FEV1, as %). SMD = standardized mean difference.

**FIGURE 6:**
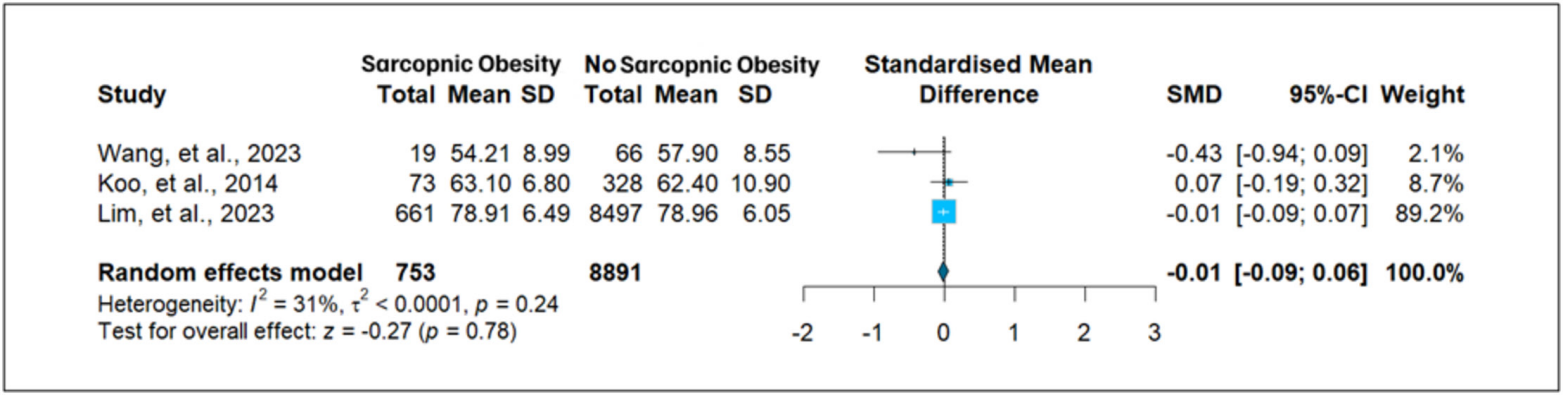
Forest plot comparing participants with and without sarcopenic obesity for the ratio of Forced Expiratory Volume in the first second (FEV1, in L) to Forced Vital Capacity (in L), expressed as %. SMD = standardized mean difference.

The sensitivity analysis showed that the level of obesity significantly modified the association between having sarcopenic obesity and FEV1 (Figure 7) the FEV1 was significantly lower in those with sarcopenic obesity compared to those without sarcopenic obesity in studies using fat mass (%) (SMD −1.14, [CI −1.21, −1.06]), whereas in studies using BMI (kg/m^2^), FEV1 was not significantly different between those having sarcopenic obesity or not (SMD −0.62, [CI −1.14, 0.17]).

**FIGURE 7:**
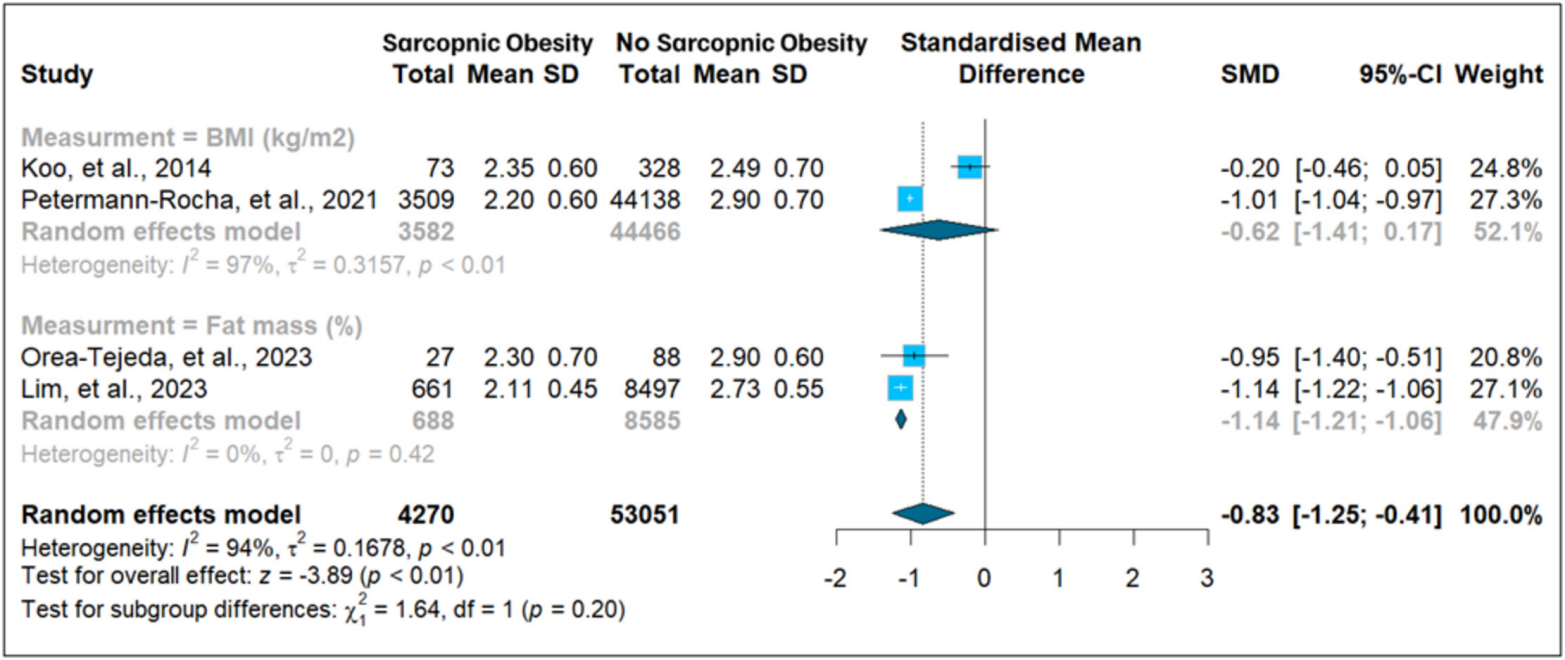
Sensitivity analysis comparing participants with and without sarcopenic obesity for Forced Expiratory Volume in the first second (FEV1, in L) grouped by obesity definitions. SMD = standardized mean difference. BMI = body mass index.

Overall, all the studies were considered to have a low risk of bias. In three studies [26, 27, 30], the description of study subjects and setting was unclear or absent. For one study [24], the methodology used to measure the outcomes was unclear. For two studies [25, 28], we believe that the statistical analysis was inappropriate (Table 3).

**TABLE 3:** Quality assessment of included studies.

Studies	Were the criteria for inclusion in the sample clearly defined?	Were the study subjects and the setting described in detail?	Was the exposure measured in a valid and reliable way?	Were objective, standard criteria used for measurement of the condition?	Were confounding factors identified?	Were strategies to deal with confounding factors stated?	Were the outcomes measured validly and reliably?	Was an appropriate statistical analysis used?
Wang et al. [24]	Yes	Yes	Yes	Yes	Yes	Yes	Unclear	Yes
Koo et al. [25]	Yes	Yes	Yes	Yes	Yes	Yes	Yes	No
Orea‐Tejeda et al. [26]	Yes	No	No	Yes	Yes	Yes	Yes	Yes
Petermann‐Rocha et al. [27]	Yes	Unclear	Yes	Yes	Yes	Yes	Yes	Yes
Moon et al. [28]	Yes	Yes	No	Yes	Yes	Yes	Yes	No
Lim et al. [29]	Yes	Yes	Yes	Yes	Yes	Yes	Yes	Yes
Zhu et al. [30]	Yes	No	No	Yes	Yes	Yes	Yes	Yes

## 4 Discussion

This systematic review and meta‐analysis evaluated the association between sarcopenic obesity and different respiratory function outcomes, using data from seven high‐quality studies representing 173,677 adult participants from five countries. This systematic review provides evidence that sarcopenic obesity is associated with significantly lower FVC and FEV1, thus indicating reduced respiratory function in those with sarcopenic obesity compared with those without sarcopenic obesity.

This is the first systematic review to consider the relationship between both sarcopenia and obesity with respiratory function. A previous meta‐analysis found a reduction in FVC of 140 mL, and in FEV1 of 150 mL, in adults with overweight/obesity alone [31], which is much more modest than the reductions of 770 and 840 mL, respectively, we found in our meta‐analysis for adults with both sarcopenia and obesity. Another systematic review and meta‐analysis found that the prevalence of sarcopenia alone was twice as high in COPD patients compared with a healthy elderly population, with a pooled prevalence of 21.6% (95% CI 14.6%–30.9%) [5]. In our analyses, we showed that having both sarcopenia and obesity gives a significantly higher risk of respiratory function impairment than in those without sarcopenia and obesity alone. Multiple factors could be responsible for this association between sarcopenic obesity and reduced respiratory function.

Respiratory muscle strength is closely related to skeletal muscle mass. Age‐related reductions in skeletal muscle mass and increased adiposity can impact the function of the respiratory muscle, leading to impaired respiratory function and respiratory complications [32, 33]. Elevated adiposity may also induce a pro‐inflammatory condition, adversely affecting both skeletal and respiratory muscle performance [34]. Furthermore, in sarcopenic obesity, skeletal muscle often exhibits fat infiltration, known as myosteatosis [35]. This could be a risk factor for the transition to severe illness in respiratory patients, such as COVID‐19 [36]. Excess adipose tissue and diminished muscle strength may hinder respiratory mechanics and ventilatory capacity [34]. Conversely, prolonged respiratory insufficiency may restrict physical activity, exacerbate inflammation, and lead to muscle atrophy and fat accumulation, suggesting a potentially bidirectional relationship [37]. Currently, we have limited knowledge of the relationship between increased fat mass and decreased muscle mass and their impact on respiratory function in asymptomatic adults without known respiratory disease [28, 38]. The definitions of sarcopenic obesity used in the studies included in the meta‐analysis were able to differentiate the impact of sarcopenic obesity on respiratory function outcomes between studies. Our study found that the significant association between sarcopenic obesity and respiratory function outcomes varied depending on the methodology used to diagnose obesity. When obesity was assessed by fat mass (%), the results remained significant; however, when using BMI, it led to a lower ability to diagnose obesity, possibly due to its inability to differentiate lean mass [39].

All studies measured spirometry outcomes to assess lung function as an outcome. One study found that patients with sarcopenic obesity had higher CAT and mMRC scores, leading to more severe dyspnea than other patients. In addition, participants with sarcopenic obesity had higher SGRQ activity, impact, symptoms, and total scores, leading to a worse quality of life than those without sarcopenia [24]. One study found that patients with sarcopenic obesity and mild, severe, and very severe COPD had a more limited airflow [25]. However, participants with sarcopenic obesity and moderate COPD had a higher airflow than participants with normal body weight [25]. Another study found that participants with sarcopenic obesity had significantly lower rates of MEF (25%–75%) PEF, DLCO, and significantly less walking distance in the 6MWT than those with normal body weight [26]. A study found that sarcopenic obesity was associated with a higher risk of respiratory disease incidence (hazard ratio: 1.51 [95% CI: 1.30 to 1.77]), but not with respiratory mortality [27]. The final study reported that the prevalence of sarcopenic obesity increased the likelihood of hospitalization in those infected with COVID‐19 more than overweight or obesity alone [30]. Assessing respiratory function in individuals with sarcopenic obesity could be a clinically relevant approach, especially now that we and others [40, 41, 42] have found evidence of an association between sarcopenic obesity and reduced lung function and increased respiratory risk. Although advanced pulmonary function testing may not be easily available in all clinical settings, simple and widely accessible instruments such as spirometry, which is widely used in primary care, could be a cost‐effective tool for routine monitoring [43]. This approach aligns with current trends in integrated care models for older adults and individuals with chronic conditions and is reported by recent findings from population‐based and COPD‐specific research [44, 45, 46].

There is evidence that the relationship between sarcopenic obesity and respiratory functions differs between men and women. Almost all included studies had male and female participants [24, 26, 27, 28, 29], except one study, which only included men [25]. Only one study provided data on sarcopenic obesity and lung function outcomes stratified by sex. The study found a significant association between sarcopenic obesity and lower FEV1 and FVC in men, but not in women [28]. However, this study did not report on the prevalence of sarcopenic obesity; thus, the effect size could not be calculated. Not all studies included in the meta‐analysis stratified for sex [24, 25, 26, 27, 29]. Sex differences in body composition and respiratory function outcomes could affect the relationship between sarcopenic obesity and respiratory outcomes [47, 48]. Men and women have different cut‐off points for sarcopenic obesity [24, 26, 27, 28, 29]. Men have more lean mass, while women have more fat mass [49]. In addition, women were found to have a higher prevalence of sarcopenic obesity than men [28, 49]. There are also sex differences in respiratory function [50]. Ordinary breathing in males and females differs, where men breathe mainly through their diaphragm, while women breathe mainly through their ribs [50]. Men also have larger absolute lung volume and volume variations than women [51]. Thus, there are different average values in healthy men and women for FVC (4.75 and 3.25 L, respectively) and FEV1 (3.5 and 2.5 L, respectively) [52]. Therefore, the relationship between sarcopenic obesity and respiratory function could be stronger in men than in women [28], but this could not be explored in the current systematic review and meta‐analysis.

The strengths of this review include the inclusion of studies with low risk of bias and the timeliness of assessing the association between sarcopenic obesity and respiratory function in adults. Examination of the spread and distribution of the effect sizes, including smaller studies with null results (Figures 2, 3, 4, 5, 6), suggests that there was no evidence of publication bias. Limitations of this systematic review include the lack of stratified outcomes on sarcopenic obesity and respiratory outcomes for men and women in most studies [24, 26, 27, 29]. In addition, studies included in the meta‐analysis [24, 25, 26, 27, 29] did not report the prevalence of sarcopenic obesity for different definitions. It is likely that differences in ways of defining and measuring sarcopenia and obesity between studies, as well as differences in study designs and target populations (e.g., male versus female participants), contributed to the high heterogeneity found in this meta‐analysis, which may make the results less interpretable. Heterogeneity requires a random‐effects meta‐analysis [53], and that is the approach we took in this study. Another limitation of this systematic review is that the data used were only obtained in five countries, which may limit the applicability of the results to other populations. Finally, all studies in the meta‐analysis were cross‐sectional, which means that it is difficult to establish a cause‐and‐effect relationship. Furthermore, the association between sarcopenic obesity and reduced respiratory function is likely bidirectional. Reduced muscle mass and increased fat mass may lead to respiratory decline, while at the same time, persistent respiratory impairment may promote muscle loss and fat accumulation. This complexity underscores the need for longitudinal studies to elucidate causality and identify of underlying biological mechanisms.

Based on this comprehensive systematic review and meta‐analysis, we can conclude that sarcopenic obesity is associated with significantly reduced respiratory function in adults. A simultaneous loss of muscle mass and increased fat mass could lead to respiratory function impairment, such as a reduction in FVC and FEV1. Sarcopenic obesity and respiratory diseases independently increase the risk of mortality; thus, having both conditions simultaneously is likely to lead to an even higher risk of mortality. Therefore, addressing both sarcopenic obesity and impaired respiratory function could have a significant impact on public health and promote healthy aging. A comprehensive assessment of respiratory function should be advised for those with sarcopenic obesity, especially in high‐risk populations such as older adults.

## Funding

This work was supported by Rural and Environment Science and Analytical Services Division (RI‐B5‐06).

## Conflicts of Interest

The authors declare no conflicts of interest.

## Supporting information


**Table S1:** Search strategy for MEDLINE (from 1946 to July 2024) and EMBASE (from 1974 to July 2024).

## Data Availability

The data that support the findings of this study are published and publicly available in the publications selected for this systematic review.
