# Supplementary material for: The Association of Sarcopenic Obesity With Respiratory Function Outcomes in Adults: A Systematic Review and Meta‐Analysis
Source: Obes Rev. 2026 May 8;27(12):e70157. doi: 10.1111/obr.70157 (PMC13749326; doi:10.1111/obr.70157)
Supplement: Supplementary file 1 [file OBR-27-e70157-s001.pdf]

The association of sarcopenic obesity with respiratory function outcomes in adults: a systematic review and meta-analysis

Tasneem Baabbad,[1] Magaly Aceves-Martins,[1] Graham Horgan,[2] Phyo Kyaw Myint,[3] Baukje de Roos[1]

[1]*The Rowett Institute, University of Aberdeen, Aberdeen, United Kingdom*
[2]*Biomathematics and Statistics Scotland, University of Aberdeen, Aberdeen, United Kingdom*
[3]*Institute of Applied Health Sciences, University of Aberdeen, Aberdeen, United Kingdom*

Contact: Baukje de Roos, corresponding author: b.deroos@abdn.ac.uk

Table S1: Search strategy for MEDLINE (from 1946 to July 2024) and EMBASE (from 1974 to July 2024)

#	Query	Results
1	(sarcopenia or Muscle atrophy or Muscle weakness or Muscle, Skeletal or Lose muscle mass or low fat free mass or handgrips or decrease muscle strength or gait speed or Skeletal muscle decline or Loss of lean body mass or loss appendicular muscle mass).af.	374,670
2	(obesity or high fat mass or body weight or high body mass index or gain weight or visceral obesity or Abdominal obesity or Hyperadiposity or overweight or Excessive body weight).af.	2,161,983
3	(Respiratory Disease or Respiratory Tract Disease or Respiratory System Diseases or Bronchitis or Bronchitis, Chronic or Chronic Bronchitis or Chronic Obstructive Bronchitis or Eosinophilic bronchitis or emphysema or lung functions or Lung emphysema or Subcutaneous emphysema or Mediastinal Emphysema or Pulmonary Emphysema or Asthma or asthma-chronic obstructive or pulmonary disease overlap syndrome or eosinophilic asthma or intrinsic asthma or mild intermittent asthma or mild persistent asthma or moderate persistent asthma or nocturnal asthma or occupational asthma or severe asthma or severe persistent asthma or cardiac asthma or extrinsic asthma or coronavirus disease 2019 or COVID-19 or Post-Acute COVID-19 Syndrome or Coronavirus or Human coronavirus 229E or Middle East Respiratory or Syndrome Coronavirus or Severe acute respiratory or syndrome-related coronavirus or Coronavirus NL63, Human or Coronavirus OC43, Human or Chronic obstructive pulmonary disease or lung Diseases or airway disease).af.	2,135,044
4	(body composition change or age related body comopsition or body composition imbalance).af.	389
5	remove duplicates from 4	254
6	(Old Population or Elderly or Elderly people or Old adult or aging or aged or adults or older adults or older people).af.	14,145,758
7	1 and 2	37,263
8	5 or 7	37,502
9	3 and 6 and 8	768
10	remove duplicates from 9	667
